# Efficacy of Cognitive Behavioral Therapy–Based Digital Therapeutics for Substance Use Disorder: Protocol for a Randomized Controlled Trial

**DOI:** 10.2196/85458

**Published:** 2026-08-07

**Authors:** Yong Chan Jeong, Byoung-Kwon Lee, Jae-Kyoung Shin, Ye-Jin Jang, Do hoon Kwon, Tae Kyung Lee, Boung Chul Lee, Haemin Seo, Ha Yeong Choi, Sang-Kyu Lee

**Affiliations:** 1 Department of Biomedical Science, Hallym University, Chuncheon Sacred Heart Hospital, Chuncheon, Republic of Korea; 2 Department of Electronic and Telecommunications Engineering, UBIPLUS Co Ltd, Chuncheon, Republic of Korea; 3 Department of Physics, Seoul National University, Seoul, South Korea; 4 Department of Biomedical Engineering, Catholic University of Daegu, Daegu, South Korea; 5 Department of Psychiatry, Daedong Hospital, Daegu, Republic of Korea; 6 Department of Psychiatry, Bugok National Hospital, Changnyeong-Gun, Republic of Korea; 7 Department of Psychiatry, Hallym University, Hangang Sacred Heart Hospital, Seoul, Republic of Korea; 8 Department of Substance Abuse and Addiction Treatment, Bugok National Hospital, Changnyeong-Gun, Republic of Korea; 9 Department of Psychiatry, Hallym University, Chuncheon Sacred Heart Hospital, 77 Sakju-ro, Chuncheon-si, Gangwon-do, Chuncheon, 24253, Republic of Korea, 82 01064451057

**Keywords:** substance use disorder, digital self-care device, abstinence success rate, superiority test, logistic regression

## Abstract

**Background:**

Illicit drug use has been rapidly increasing in South Korea, particularly among individuals in their 20s, contributing to a growing burden of substance use disorder (SUD). However, treatment infrastructure remains limited. Nationwide, only a small number of inpatient treatment hospitals are available, and treatment capacity in the Seoul metropolitan area is insufficient to meet growing demand. In addition, community-based addiction services are scarce, creating barriers to continuous care. Digital therapeutics (DTx) have emerged as a promising approach to improve treatment accessibility and continuity of care. In particular, cognitive behavioral therapy (CBT)–based DTx such as RESET-O, developed in the United States and authorized by the US Food and Drug Administration, have demonstrated clinical benefits in supporting addiction recovery. Building on this concept, our team developed D-STOP, a CBT-based DTx intervention designed to support individuals with SUD in the Korean clinical context.

**Objective:**

This study aims to evaluate the efficacy of D-STOP as an adjunctive DTx intervention for patients with SUD.

**Methods:**

This study is a randomized controlled clinical trial designed to evaluate the efficacy of D-STOP in individuals diagnosed with SUD. Following an initial screening assessment, 118 participants meeting the diagnostic criteria will be enrolled. Participants will be recruited from psychiatry departments at addiction treatment and clinical care institutions in Chuncheon, Seoul, Daegu, and Changnyeong, South Korea. During the 12-week intervention period, the experimental group will receive D-STOP in addition to treatment as usual, whereas the control group will receive treatment as usual alone. D-STOP delivers structured CBT-based modules and motivational enhancement interventions through a digital platform. During scheduled study visits, participants will also receive therapeutic feedback from psychiatrists or trained study staff. The primary end point is the abstinence success rate during weeks 9 to 12 of treatment. Logistic regression analysis will be used to estimate treatment effects and evaluate superiority compared with the control group.

**Results:**

The study protocol was approved by the institutional review board of Hallym University Chuncheon Sacred Heart Hospital on April 14, 2025. Funding began on April 1, 2023. Data collection started on August 4, 2025, and is expected to be completed by November 30, 2026. As of March 6, 2026, a total of 65 participants have been enrolled. An interim analysis of the primary efficacy outcome has been conducted based on the data available at the time of analysis; however, statistical significance has not been established due to the limited sample size. The final analysis is expected to be published in April 2027.

**Conclusions:**

DTx have the potential to expand access to evidence-based addiction treatment in resource-constrained settings. This study will provide clinical evidence on the efficacy and feasibility of D-STOP as a digital treatment support tool for patients with SUD.

## Introduction

In South Korea, the spread of narcotics has been accelerating. In particular, the proportion of overdose cases among individuals in their 20s has increased markedly over the past several years, accounting for more than 30% of all identified cases and representing the largest affected age group. Among young adults with mental health problems, a concerning cycle has emerged in which individuals seek psychotropic substances and subsequently engage in repeated misuse. As a result, many addiction specialists have emphasized the urgent need for comprehensive measures to promote treatment engagement and prevent relapse, including policy initiatives, expansion of treatment services, and the development of recovery and rehabilitation programs [1].

Despite the increasing prevalence of substance use disorder (SUD), treatment infrastructure in South Korea remains limited. Only a small number of hospitals provide specialized inpatient treatment for individuals with SUD, and access to intensive treatment programs is restricted in many regions [2]. In the Seoul metropolitan area, where a large proportion of drug-related crime occurs, the availability of treatment facilities remains insufficient to meet the growing demand. Furthermore, community-based addiction services remain underdeveloped, limiting opportunities for continuous treatment and long-term recovery support [3]. Social stigma associated with drug use also creates additional barriers to care, discouraging individuals from seeking treatment and contributing to delayed intervention [4].

The challenges surrounding addiction treatment are not unique to South Korea. There is international evidence suggesting that a large proportion of individuals with SUD do not receive adequate treatment. For example, national survey data from the United States indicate that many individuals with SUD do not access treatment services due to structural barriers such as limited treatment availability, financial constraints, or lack of motivation for treatment engagement [5]. In addition, structural inequalities in health care systems may further restrict access to addiction treatment and medication-assisted therapy in socially disadvantaged communities [6].

Psychosocial interventions remain the cornerstone of treatment for SUD. Among these approaches, cognitive behavioral therapy (CBT) has consistently demonstrated effectiveness in reducing substance use and preventing relapse across numerous clinical trials and meta-analytic studies [7-10]. CBT focuses on identifying maladaptive cognitive patterns and behavioral triggers associated with substance use while strengthening the coping strategies and self-regulation skills necessary for sustained recovery [11,12].

In addition to CBT, motivational enhancement therapy (MET) and motivational interviewing techniques are widely used to address ambivalence toward behavior change in patients with addiction [13,14]. These approaches aim to strengthen individuals’ intrinsic motivation for recovery and improve engagement in treatment. Previous studies have shown that motivational interviewing can significantly enhance readiness for change and treatment adherence, particularly during the early stages of addiction treatment [15,16].

More recently, mindfulness-based interventions have been introduced as complementary approaches for managing craving and emotional dysregulation associated with SUD [17]. Mindfulness-based relapse prevention programs aim to increase awareness of craving, emotional distress, and automatic behavioral responses related to substance use [18]. There is evidence suggesting that mindfulness-based interventions can reduce relapse risk by improving emotional regulation and attentional control, thereby enabling individuals to respond more adaptively to craving and stress-related triggers [19].

In addition, stress management and relaxation-based interventions have been incorporated into addiction treatment programs to address the role of stress in relapse vulnerability. Stress exposure has long been recognized as a critical factor contributing to craving and relapse among individuals with substance dependence. Techniques such as breathing training, progressive muscle relaxation, and guided relaxation exercises may reduce physiological arousal and improve coping responses to stress-related triggers [20,21].

Digital therapeutics (DTx) have emerged as promising tools for delivering evidence-based behavioral interventions at scale, particularly in areas where access to specialized addiction treatment services is limited. Advances in digital health technologies have recently enabled these evidence-based psychological interventions to be delivered through DTx platforms. DTx provide structured behavioral treatment through software-based programs that can be accessed remotely, allowing patients to engage in therapeutic interventions outside traditional clinical settings. Such approaches have the potential to improve treatment accessibility, enhance continuity of care, and reduce barriers related to stigma or geographical limitations [22,23].

One example is RESET-O, a prescription DTx intervention authorized by the US Food and Drug Administration for the treatment of SUD [24]. The effectiveness of digital behavioral interventions for SUD has also been supported by randomized controlled trials evaluating internet-delivered treatments [25].

Building on these treatments, our research team developed D-STOP, a CBT-based DTx intervention designed to support individuals with SUD in South Korea. In addition to CBT-based treatment modules, D-STOP incorporates complementary psychological strategies, including motivational enhancement, mindfulness-based coping techniques, psychoeducation, and crisis de-escalation strategies.

This study evaluates the efficacy, validity, and acceptability of D-STOP as a DTx intervention for individuals with SUD. By enabling patients to engage in structured behavioral treatment remotely, the intervention may help overcome barriers related to stigma, limited treatment access, and insufficient treatment infrastructure in South Korea.

## Methods

### Aim

The primary objective of this confirmatory clinical trial is to evaluate the feasibility, therapeutic efficacy, and acceptability of a CBT-based DTx intervention (D-STOP) within Korean health care settings. Feasibility and efficacy will be assessed by determining whether the use of D-STOP, administered as an adjunct to treatment as usual (TAU) for patients with SUD, leads to improvement in addiction-related symptoms.

### Design

Using a prospective design, we will collect longitudinal data on patients with SUD for subsequent analysis. Participants will be assigned in a 1:1 randomized fashion to a control group (TAU) or an experimental group (TAU+D-STOP). The trial will be conducted in an open-label manner. The primary aim is to demonstrate the superiority of the experimental arm over the control arm in treatment efficacy. Outcomes will be derived by integrating quantitative and qualitative data sources, including measures captured within the D-STOP app and standardized diagnostic and assessment instruments.

The meaning and implementation of TAU may vary across different medical specialties. In this study, TAU refers to maintaining the medications and treatment approaches that participants were receiving prior to enrollment in the clinical trial. With respect to medications, participants are allowed to reduce the dosage or discontinue previously prescribed medications during the study period; however, increasing the dosage or introducing new medications is not permitted.

Regarding psychosocial treatments, participants engaged in psychosocial intervention programs prior to enrollment (eg, Narcotics Anonymous meetings or 12-step addiction recovery programs) are allowed to continue participating in them. However, the initiation date of the psychosocial treatment program is required to precede the start date of participation in this clinical trial. To minimize potential confounding effects, preference is given to enrolling individuals who are not actively participating in such programs.

In addition, TAU in this study is limited to patients receiving outpatient care only. During the clinical trial period, the total number of hospital visits is scheduled to be 7. If a participant receiving outpatient care requires hospitalization during the trial period, this is considered a relapse-related event and recorded as a notable clinical occurrence; however, such cases are not classified as study withdrawal.

### Randomized Controlled Trial

Randomization will be conducted using an interactive web response system. Participants who meet all inclusion criteria will be assigned to either the experimental group or the control group according to a pregenerated randomization list in the order of enrollment.

The randomization list will be generated using the PROC PLAN procedure in the SAS software (version 18.0.1; SAS Institute). The block size and seed number used for the randomization sequence will be randomly determined by a statistician responsible for randomization at the clinical trial center.

Access to the randomization schedule will be restricted to the designated statistician only. Because allocation will be implemented through a centralized randomization system, the investigators will not have prior knowledge of the randomization sequence or the assignment results, ensuring allocation concealment.

Randomization will be performed after eligibility has been confirmed and the screening procedures have been completed.

### Participants

This multicenter study is being conducted at 4 medical institutions in South Korea that provide treatment for SUD. Participants will be recruited at university hospitals and general hospitals that include departments of psychiatry using both in-hospital posters and external (community-facing) advertisements.

This study aims to recruit 118 participants across 4 regions in South Korea (Seoul, Daegu, Chuncheon, and Changnyeong) based on estimates from prior research on the required sample size to demonstrate superiority (Textbox 1).

The clinical trial will last 12 weeks, with a total of 7 visits. Each visit window allows for –5 to +5 days from the scheduled date. Participants who fail to attend within this window will be considered to have discontinued the study (Table 1).

Textbox 1. **Inclusion and exclusion criteria.**

**Inclusion criteria**

- Adults aged 19 years or older
- Fulfillment of the diagnostic criteria for substance use disorder according to the *Diagnostic and Statistical Manual of Mental Disorders, Fifth Edition* [26] and diagnosis under the *International Classification of Diseases, Tenth Revision*, F11, F12, F14, F15, or F19 categories [27]
- At screening, completion of the Drug Abuse Screening Test–Korean Version [28]
- Having initiated psychiatric treatment within 30 days prior to screening and intention to continue treatment for at least 3 months
- Self-reported history of drug use within 30 days prior to screening (or within 60 days if recently discharged from a controlled environment)
- Ability to read and write Korean without difficulty
- Ability to use a smartphone and mobile app without difficulty
- Voluntary provision of written informed consent after receiving sufficient explanation of the study
- No history of dementia
- For women of childbearing age, agreement to use medically acceptable contraception during the study period
- In special cases, legally authorized representatives may consent on behalf of participants; such participants are also eligible

**Exclusion criteria**

- Presence of any advanced, progressive, or unstable disease that could endanger the patient
- Neurological disorders that may cause cognitive impairment
- Neurodegenerative diseases other than depression or severe psychiatric symptoms that would preclude study participation
- Current uncontrolled seizure disorder
- History of brain injury with loss of consciousness lasting more than 1 hour or hospitalization due to traumatic brain injury within the past 3 years
- Hearing or visual impairment that would interfere with completion of study requirements
- Lack of a smartphone capable of installing the investigational medical app
- Pregnant or breastfeeding women
- Concurrent participation in another clinical trial or participation in another trial within 30 days prior to screening
- Determination by the investigator to be otherwise unsuitable for the trial

**Table 1.** SPIRIT (Standard Protocol Items: Recommendations for Interventional Trials) checklist: clinical trial–related items.

	Clinical trial period						
	Enrollment—visit 1 (week –4)	Assignment—visit 2 (week 0; baseline)	After assignment				Completion—visit 7 (week 12)
			Visit 3 (week 4)	Visit 4 (week 6)	Visit 5 (week 8)	Visit 6 (week 10)	
Enrollment							
Informed consent	✓						
Screening	✓						
Eligibility check (inclusion and exclusion criteria)	✓						
Demographics and social history	✓						
Physical examination	✓						
Prior medication history	✓						
Pregnancy test	✓	✓	✓^a^	✓^a^	✓^a^	✓^a^	✓^a^
Urine drug test		✓	✓	✓	✓	✓	✓
Randomization		✓					
Intervention							
Investigational DTx^b^		✓	✓	✓	✓	✓	✓
TAU^c^	✓	✓	✓	✓	✓	✓	✓
Assessment							
Abstinence record sheet and mobile record verification							✓
Questionnaire assessment^d^		✓			✓		✓
Compliance and retention assessment		✓	✓	✓	✓	✓	✓
Adverse event monitoring		✓	✓	✓	✓	✓	✓

a Proceed, if applicable.

b DTx: digital therapeutics.

c TAU: treatment as usual.

d Questionnaire assessment: University of Rhode Island Change Assessment Scale–Drug Offender, Hospital Anxiety and Depression Scale, Cognitive Reappraisal Questionnaire, and Behavioral Activation for Depression Scale.

### Outcome Measures

#### Primary Outcome Measure: Abstinence Success Rate During the Previous 4 Weeks

The primary end point is the abstinence success rate during the final 4 weeks (weeks 9‐12) of the 12-week trial. Abstinence success is defined as meeting both of the following criteria: (1) testing negative on urine drug screens conducted at study visits and (2) reporting “no drug use” on the abstinence check function within D-STOP, consistently maintained throughout the final 4-week period. Participants who satisfy both conditions are classified as achieving abstinence success. The designation of abstinence during the final 4 weeks (weeks 9‐12) of the 12-week trial as the primary efficacy end point was informed by the primary efficacy end point used in the clinical trial of RESET-O.

For the control group, D-STOP is not provided. Instead, abstinence status will be monitored using a paper-based checklist to record participants’ self-reported drug use.

#### Secondary Outcome Measures

##### Abstinence Success Rate During the 12-Week Treatment Period

The proportion of participants achieving abstinence at any point during the 12-week study period will be collected. These data will inform evaluation of abstinence duration and maintenance.

##### Treatment Retention Rate During the 12-Week Treatment Period

The proportion of participants who complete the 12-week study without dropout will be assessed. Treatment retention is defined as the completion of all trial procedures without premature discontinuation. This measure is particularly important for patients with SUD, who face high relapse rates. High treatment retention itself can serve as an indicator of the feasibility and validity of the D-STOP app.

### Data Collected Within D-STOP

The following variables will be collected: (1) longest abstinence period, (2) total abstinent days, and (3) results from the following psychometric instruments.

#### University of Rhode Island Change Assessment Scale–Drug Offender (Korean Version; Drug-Related Readiness to Change Questionnaire)

Originally developed by McConnaughy et al [29] and validated in Korean by Chung et al [30], the University of Rhode Island Change Assessment Scale–Drug Offender is a 24-item questionnaire scored on a 5-point Likert scale. It comprises 3 stages (precontemplation, contemplation, and action or maintenance). Scores of 7 or lower indicate precontemplation, scores of 8 to 11 indicate contemplation, and scores of 12 or higher indicate action or maintenance. Reliability (Cronbach α) has been reported as 0.83 overall, 0.82 for precontemplation, 0.79 for contemplation, and 0.81 for action or maintenance. Construct and convergent validity indexes exceed 0.5 and 0.7, respectively, confirming strong reliability and validity.

#### Hospital Anxiety and Depression Scale (Korean Version)

On the basis of the Hospital Anxiety and Depression Scale by Zigmond and Snaith [31], the Korean version was validated by Oh et al [32]. The 14-item instrument, rated on a Likert scale from 0 to 3, consists of 7 anxiety items and 7 depression items. Cutoff scores are 7 or lower for healthy, 8 to 10 for borderline anxiety or depression, and 11 or higher for clinical anxiety or depression.

#### Cognitive Reappraisal Questionnaire

Developed by Kim [33] in South Korea, the Cognitive Reappraisal Questionnaire assesses 2 subtypes of cognitive reappraisal (objectifying and positive reappraisal). It is a validated 20-item questionnaire rated on a Likert scale from 1 to 7. The measure evaluates the frequency of specific emotion regulation strategies. Internal consistency (Cronbach α) was reported as above 0.80 for both subscales, indicating excellent reliability.

#### Behavioral Activation for Depression Scale

The Behavioral Activation for Depression Scale is a 25-item self-report questionnaire designed to assess patient activation levels and treatment response based on behavioral activation theory. It consists of 4 subscales: activation, avoidance or rumination, work or school impairment, and social impairment, each rated on a Likert scale from 1 to 7. The Korean version was validated by Oh et al [34]. Overall internal consistency of a Cronbach α value of 0.91 has been reported, with subscale Cronbach α values ranging from 0.76 to 0.90, demonstrating excellent reliability.

### Psychiatric and Psychological Intervention: CBT-Based DTx for SUD (D-STOP)

#### Overview

The DTx app used in this study was developed based on CBT. In addition, to enable patients to process information at their own pace and attempt treatment in alignment with their recovery plans, the app incorporates visual materials. These are grounded in established psychiatric and psychological approaches, including MET, psychoeducation, mindfulness, and conditioned reflex inhibition techniques, thereby providing a comprehensive therapeutic intervention.

#### Procedures

This clinical trial is currently in the recruitment phase. Recruitment strategies include conducting screening tests for patients with SUD who present at 1 of the 4 participating regional medical institutions. Eligibility will be determined based on the inclusion and exclusion criteria described above, and suitable participants will be enrolled. Additional recruitment will be carried out through community organizations (eg, the Seoul branch of the Korea Association Against Drug Abuse) using external advertisements.

During the 12-week trial period, participants will be required to attend 7 visits. At each visit, the following will be assessed: (1) abstinence status within the app, (2) treatment adherence, (3) diagnostic assessments for secondary end points, and (4) urine drug screens and pregnancy tests.

If drug relapse is detected through urine testing or through abstinence status recorded on the app during weeks 1 to 8, the participant will continue in the clinical trial. However, relapse occurring during weeks 9 to 12, which correspond to the assessment period for the primary end point, will be considered treatment failure.

With respect to treatment adherence, if participants use less than 35% of the app’s therapeutic services, the study psychiatrist or clinical research coordinator will encourage them to improve their engagement.

Upon enrollment, participants will be provided with the DTx app. Over the course of the 12-week self-management program, they will complete 17 modules accessible via the app: 6 treatment modules, 4 emergency modules, 1 daily life management module (via a calendar), and 6 relaxation training modules.

Throughout the trial, adverse events will be monitored. If an adverse event occurs, it will be documented in the adverse event log and reported to the study sponsor within 24 hours via the rapid adverse event reporting system.

#### D-STOP Intervention Process and Treatment Mechanism

D-STOP consists of 17 modules; however, each module is not limited to a single therapeutic method. Rather, an embedded algorithm dynamically provides different therapeutic components depending on participants’ responses. For example, the treatment case module includes 30 video-based materials organized into 5 thematic categories, with 6 videos assigned to each category. Other treatment modules also contain multiple therapeutic materials, and the total number of therapeutic components in the entire program, including treatment case materials, is 79.

Unlike RESET-O, this study places greater emphasis on the delivery of individual therapeutic materials rather than the number of modules completed. This design was adopted to address potential screen fatigue associated with prolonged use of app-based treatment. Accordingly, D-STOP was developed not merely as an educational program but also as an interactive therapeutic system. The visual materials presented to patients typically last approximately 1 to 2 minutes, and the program promotes multisensory engagement through tactile interaction via manual input, auditory learning through listening exercises, and vocal practice through spoken responses. This multisensory approach is intended to facilitate active participation and experiential learning.

On average, completing a single therapeutic activity requires approximately 10 minutes. Through the recommendation system embedded within the app, participants are generally encouraged to complete approximately 3 therapeutic activities per day. In addition, an acuity assessment conducted every 2 weeks identifies participants’ current clinical needs and dynamically recommends appropriate therapeutic components. Through this process, D-STOP provides a personalized treatment pathway tailored to each participant’s clinical condition. Participants are expected to engage with the intervention regularly throughout the 12-week treatment period.

#### D-STOP Modules

##### Overview

**Figure 1.**
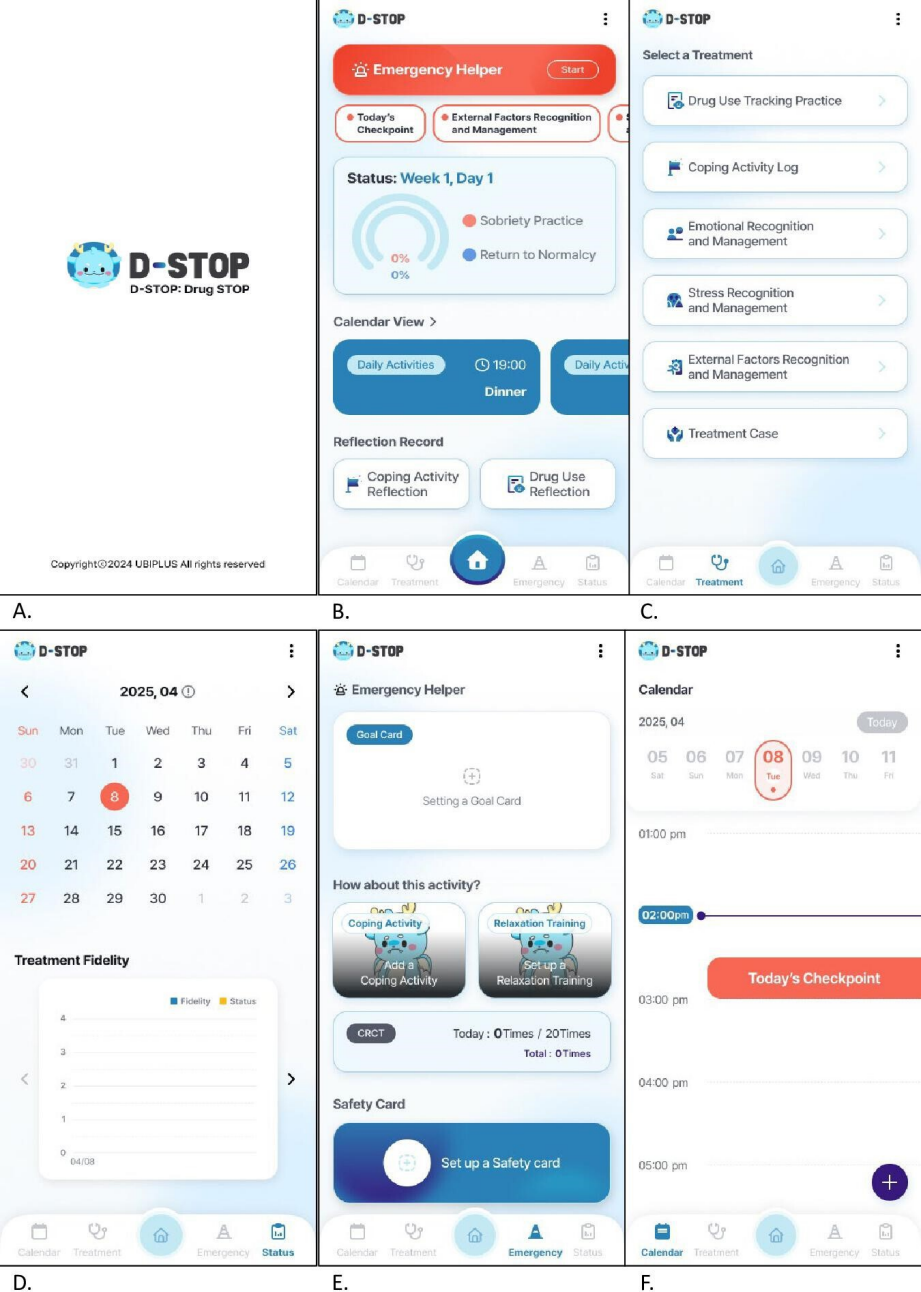
Sample screenshots of the D-STOP app: (A) main access screen; (B) home screen; (C) treatment module screen; (D) calendar screen; (E) emergency coping screen; and (F) schedule setting screen.

The modules implemented in D-STOP were not designed to simply replicate conventional psychotherapeutic approaches. Instead, they were developed with a strong emphasis on user interface and user experience to maximize patient engagement with the app while maintaining the therapeutic efficacy of established psychological interventions (Table 2 and Figure 1).

**Table 2.** D-STOP module list.

Category	Module
CBT^a^	Module 1: drug use tracking practice Module 2: emotional recognition and management Module 3: stress recognition and management Module 4: external factor recognition and management
MET^b^	Module 5: treatment case Module 6: goal cards Module 7: treatment fidelity Module 8: coping activity log
Mindfulness	Module 9: cyclical sigh breathing Module 10: the 5-4-3-2-1 grounding technique Module 11: progressive muscle relaxation Module 12: urge surfing Module 13: 5-sense mindfulness practice Module 14: silent mindful walking
CM^c^	Module 15: calendar Module 16: daily activities Module 17: drug check

a CBT: cognitive behavioral therapy.

b MET: motivational enhancement therapy.

c CM: contingency management.

##### CBT Module

The CBT module was designed differently from traditional CBT formats. Instead of focusing primarily on identifying negative thoughts (automatic thoughts) and gradually inducing behavior change through multiple sessions, the module aims to help patients quickly recognize their problems and adjust both cognition and behavior through small, daily behavioral modifications.

In the drug use tracking practice module, patients are encouraged to recall and reflect on their previous experiences of drug use. This process helps patients identify the underlying factors that contributed to their substance use within the D-STOP intervention. While completing this exercise, patients are asked to report the primary reason associated with their drug use experience. On the basis of the identified factor, the system recommends and initiates 1 of 3 follow-up modules—emotional recognition and management, stress recognition and management, or external factor recognition and management—to facilitate a more detailed exploration of the relevant triggers.

##### MET Module

Traditional MET includes a variety of approaches. However, in D-STOP, the MET module was designed not as a therapist-led counseling intervention but as a self-guided module intended to help patients identify their own motivation while participating in the program, thereby strengthening their capacity to overcome addiction.

Patients are presented with treatment case scenarios involving individuals who encountered or reused drugs due to circumstances similar to the participants’ own. By observing how these individuals eventually recovered from addiction, patients may experience a mirroring effect, which can foster a belief that they too are capable of overcoming substance dependence.

To sustain this emerging motivation, the program provides a “goal card” function through which patients can define personal reasons for recovery or identify motivational triggers that reinforce their commitment to overcoming addiction. In addition, to encourage lifestyle changes, the app recommends alternative activities that can be easily scheduled within a calendar system. This approach supports contingency-based behavioral management and promotes continued engagement with recovery-oriented behaviors.

Furthermore, as patients consistently participate in the program, a visual treatment progress curve is presented within the app. The upward trajectory of this progress indicator helps patients recognize their gradual recovery from addiction and improvements in quality of life, thereby reinforcing their motivation to continue treatment.

##### Mindfulness Module

Finally, the mindfulness component was developed in a manner closely aligned with traditional mindfulness practices and implemented in a format that patients can easily observe and follow. This module introduces 6 relaxation-based techniques designed to help patients manage and reduce cravings associated with substance use.

The techniques are presented in two formats: (1) step-by-step guided exercises that can be practiced gradually and (2) rapid relaxation strategies that can be applied immediately when cravings become intense.

These practices aim to help patients regulate craving-related distress and restore psychological stability through focused attention and sensory awareness.

### Data Analysis

All data collected in this trial will be analyzed based on the entire screened population. The number of participants excluded from the safety set and the reasons for exclusion will be reported relative to all enrolled participants. Exclusions from the full analysis set will be described relative to the safety set, and exclusions from the per-protocol set will be described relative to the full analysis set. The primary efficacy analysis will be conducted on the full analysis set according to the intention-to-treat principle.

Participants who discontinue the study prematurely (dropouts) will be included in the analysis in accordance with the intention-to-treat principle. Missing outcome data will not be imputed. If missing values occur in outcomes related to abstinence status, the participant will be considered as having failed (failure) for the purpose of analysis.

For efficacy analyses, the primary end point is the abstinence success rate during the final 4 weeks of the trial. This end point is intended to evaluate the efficacy of the DTx intervention in patients with SUD. Statistical significance will be assessed using multiple logistic regression analyses adjusting for stratification factors such as stimulant vs nonstimulant drug use and baseline abstinence status (abstinent vs nonabstinent) as covariates. The treatment effect will be expressed as the adjusted odds ratio for the experimental group relative to the control group along with 2-sided 95% CIs.

Superiority of the experimental group over the control group will be concluded if both of the following criteria are satisfied: (1) the 2-sided *P* value is less than .05, and (2) the lower bound of the 2-sided 95% CI for the adjusted odds ratio exceeds 1.0.

The secondary efficacy end points include (1) abstinence maintenance rate during the 12-week treatment period, (2) treatment retention rate during the 12-week treatment period, (3) total number of abstinent days, (4) longest duration of consecutive abstinent days, and (5) results from psychiatric and psychological assessment tools.

For these outcomes, multiple logistic regression analyses and analyses of covariance will be performed to compare differences between the experimental and control groups.

### Ethical Considerations

This study was reviewed and approved by the institutional review board (IRB) of Hallym University Chuncheon Sacred Heart Hospital (CHUNCHEON 2025-03-004-004) and has been formally registered with the Clinical Research Information Service of the Korea Disease Control and Prevention Agency, a World Health Organization–recognized primary registry (registration KCT0010968).

The research team will provide each participant with a detailed explanation of the clinical procedures, expected treatment effects, and therapeutic services provided. This explanation will take approximately 30 minutes, after which written informed consent will be obtained. Participants will then receive access to the DTx app, followed by an orientation session on its use, which will also last approximately 30 minutes. Therefore, the total explanation and orientation period will be of approximately 1 hour.

During the course of the trial, each participant will be assigned a unique study identification number, which will be recorded in the electronic case report form for monitoring and data management purposes. All data collected during the study will remain confidential and will be securely stored in accordance with the IRB-approved protocol.

As the study involves no invasive procedures and the investigational DTx app is noninvasive, no serious risks are anticipated; therefore, no independent data monitoring committee has been established, as approved by the IRB. Nevertheless, internal investigators will continuously monitor participants’ treatment progress throughout the trial.

Participants enrolled in the D-STOP clinical trial will receive modest compensation for their participation. Compensation is provided at 3 time points across the 7 scheduled study visits (including the enrollment visit): at the baseline visit, the fifth visit, and the final seventh visit. Unlike the contingency management approach used in RESET-O, these incentives are not delivered immediately in response to treatment adherence or abstinence outcomes. Instead, they serve solely as nominal compensation for participation in the clinical study. Each compensation is KRW 50,000 (US $35) per visit, and participants will receive a total compensation of KRW 150,000 (US $105).

## Results

The study has been funded for a 3-year period starting on April 1, 2023. This study has progressed to the initiation of the confirmatory clinical trial following approval from the Ministry of Food and Drug Safety and an IRB amendment review at Hallym University Chuncheon Sacred Heart Hospital in April 2025. Data collection began on August 4, 2025, and is expected to be completed by November 30, 2026. As of March 6, 2026, a total of 65 patients have been enrolled, and an interim analysis has been conducted based on the data available at the time of analysis. However, statistical significance could not be established due to the limited sample size. A final analysis will be conducted once all data have been collected, and the results are expected to be reported in spring 2027.

## Discussion

The aim of this study is to evaluate the efficacy, acceptability, and feasibility of a CBT-based DTx intervention for individuals with SUD. Previous studies have demonstrated that CBT provides significant therapeutic benefits for patients with SUD, and RESET-O—the first DTx intervention of its kind—showed considerable success in its confirmatory clinical trial. Furthermore, the dissemination of DTx as a means of delivering remote treatment is highly beneficial for patients with SUD in South Korea. Although RESET-O ultimately faced limitations in the United States due to insurance-related issues, the Korean health care environment provides a more favorable setting: insurance barriers are comparatively easier to resolve, and patients do not face heavy financial burdens in accessing therapeutic devices. Unlike RESET-O, which primarily relied on one-way delivery of CBT educational videos and workbooks, D-STOP has the advantage of an interactive treatment model that enables patients to build personalized treatment behavior patterns through engagement with the program. In addition, D-STOP equips patients with immediate coping strategies to reduce cravings and improve daily lifestyle management, thus offering a wider range of therapeutic options.

Despite these strengths, this study has several limitations. First, the DTx format may increase the burden on patients. In traditional face-to-face CBT, the therapeutic effect often depends on the clinician’s level of expertise—sometimes alleviating patient burden—whereas DTx place more responsibility on patients themselves. However, because CBT can be ineffective for some patients when clinicians fail to explain concepts clearly, we addressed this limitation by simplifying CBT terminology within D-STOP to ensure accessibility and comprehension. A usability evaluation was also conducted to confirm that patients could easily understand and use the intervention.

Another limitation is that participants were not stratified by severity of SUD. Clinicians often consider even using drugs a single time as an indicator of severe clinical risk. For research purposes, ideally, treatment efficacy should be validated across more finely stratified subgroups; however, recruiting participants with SUD in South Korea presents practical difficulties. Unlike in the United States or other countries where research and treatment for SUD are more advanced, South Korea is only beginning to initiate systematic clinical research in this field, and drug use has only recently emerged as a major social issue. Nevertheless, this study may serve as a foundation for future research on SUD treatment in South Korea, laying the groundwork for subsequent studies to be conducted with greater precision and more rigorous methodologies.

## Abbreviations

CBT: cognitive behavioral therapy
DTx: digital therapeutics
IRB: institutional review board
MET: motivational enhancement therapy
SUD: substance use disorder
TAU: treatment as usual
